# Forever Chemicals, Finite Defenses: PFAS Burden the Liver, Break Mitochondria, and Outpace Modern Regulation

**DOI:** 10.3390/ijms27062723

**Published:** 2026-03-17

**Authors:** Aarush Goyal, Melike Kesmez, Nukhet Aykin-Burns

**Affiliations:** 1 Division of Radiation Health, Department of Pharmaceutical Sciences, College of Pharmacy, University of Arkansas for Medical Sciences, Little Rock, AR 72205, USA; agoyal@uams.edu; 2 Faculty of Pharmacy, Ataturk University, Ataturk Mah. Yakutiye, Erzurum 25240, Türkiye; melike.kesmez22@ogr.atauni.edu.tr

**Keywords:** PFAS, hepatotoxicity, liver bioaccumulation, mitochondrial dysfunction, emerging PFAS, environmental health regulation

## Abstract

Per- and polyfluoroalkyl substances (PFAS) continue to be one of the most persistent global contaminants and are increasingly recognized as leading metabolic- and hepatic-dysfunction mediators. Despite extensive investigation of PFAS toxicity, a critical gap in the identification and integration of toxicokinetic drivers of hepatic bioaccumulation with mechanistic pathways driving mitochondrial and nuclear receptor-related injury, more specifically, with respect to alternative PFAS strategies, still remains. Legacy PFAS, including PFOA and PFOS, accumulate in the liver and disturb mitochondrial homeostasis as they disrupt β-oxidation, induce oxidative stress, and alter lipid and bile acid metabolism. Meanwhile, the next-generation PFAS variants (including short-chain and polymeric substitutes) are rapidly increasing in environmental concentrations, but remain insufficiently characterized and poorly regulated, raising concerns that substitution-based strategies may maintain their toxicological risk. We summarize the evidence of the association between PFAS bioaccumulation and mitochondrial dysfunction, metabolic reprogramming, and inflammatory signaling, and illustrate mechanistic convergence across legacy and emerging PFAS. We also review insights from recent experimental models, such as 3D hepatocyte systems and human-relevant receptor platforms that more closely mimic chronic exposure states. This review emphasizes mechanistic convergence across legacy and emerging PFAS, highlighting shared pathways that may persist despite chemical substitution. Thus, we discuss key gaps in monitoring, toxicity assessment, and policy, including the requirement of regulatory paradigms that treat PFAS as a class rather than individual compounds.

## 1. Introduction

Per- and polyfluoroalkyl substances (PFAS), or “forever chemicals”, are synthetic substances widely used in industrial and consumer products. The compounds are found in food packaging, pesticides, firefighting foams, waterproofing products, paints, and cosmetics [1,2]. Environmental studies have shown PFAS buildup in terrestrial and marine systems and processed foods [3,4,5,6,7]. Uptake of PFAS into edible crops grown on biosolids-amended soils is now a well-documented additional dietary pathway [8]. Beer and tea have also emerged as unexpected sources of ultrashort-chain PFAS such as trifluoroacetate [9].

Such broad exposure results in substantial human exposure, primarily through dietary consumption. Since PFAS compounds have caused significant environmental contamination, understanding how they enter the human population is essential for proper health risk assessment. The application of diverse PFAS compounds in food-contact materials is a significant concern. These are applied to food packaging containers and nonstick cookware [10,11]. The transport of PFAS compounds from these materials can be a major route for food contaminants and human exposure through dietary consumption [12,13]. Beyond individuals exposed to these contaminants, some occupational categories are exposed to significantly higher levels. Firefighters, who are constantly exposed to aqueous film-forming foams and industrial workers in fluorochemical manufacturing, have substantially higher PFAS exposures [14,15]. These occupational exposures may help clarify the health impacts of higher PFAS doses, complementing our knowledge from population-level studies.

## 2. Global Epidemiological Evidence of Widespread PFAS Exposure

Per- and polyfluoroalkyl substances (PFAS) are a public health concern worldwide, and human exposure is reported globally, as on many continents. Biomonitoring data from the United States National Health and Nutrition Examination Survey (NHANES) confirm nearly uniform exposure, with detection rates above 98% of legacy PFAS compounds (PFOS, PFOA, PFHxS, PFNA) in the U.S. general population since 1999 [16,17]. However, over 96% of U.S. adolescents aged 12–19 years have detectable serum concentrations of these legacy compounds [17], despite significant drops in geometric mean concentrations of PFOS (more than 85%) and PFOA (more than 70%) between 1999–2000 and 2017–2020 as industrial phase-outs took place. European biomonitoring using the Human Biomonitoring for Europe (HBM4EU) effort (2017–2022) supports this extensive exposure pattern, with the most common PFOA, PFOS, PFHxS and PFNA detected at above 95% prevalence amongst European teenagers and 14.3% of all adolescents studied exceeding the EFSA health-based guidance value of 6.9 μg/L for the sum of four PFAS, with rates as high as 24% in Northern and Western Europe [18,19]. The same pattern of global and regional exposure is recorded with East Asian populations: detection rates were greater than 99.7% in five of the most prevalent PFAS evaluated by South Korea’s National Biomonitoring Programme (KoNEHS cycle 4, 2018–2020), and geometric mean serum PFOS concentrations were 15.1 μg/L in adults and 7.97 μg/L in adolescents, which are larger compared to those of contemporary North American and European populations [20]. Biomonitoring studies in Japan suggest elevated exposure to PFAS (16.1–43.5 ng/mL total PFAS) in contaminated spaces near military bases and fluorochemical sites used in production, which indicate significant populations exposed to the PFAS based on established risk assessment guidelines [21]. High-exposure populations located in the vicinity of areas near industrial point sources bear tremendous burdens: communities closer to AFFF-contaminated sites near U.S. military installations had concentrations of serum PFOS, PFOA, and PFHxS 2–10 times higher than national reference levels years after drinking water exposures were remediated [17]. Furthermore, European data on occupational exposures showed heightened contamination levels in chrome plating facility workers and surrounding fluorochemical manufacturing hotspots with exceedance rates as high as 23.8% higher than health-based guidance values [19]. Collectively, this epidemiological data creates accumulating proof that PFAS exposure is nearly ubiquitous among industrialized populations and persistence of legacy compounds are still far above the endpoint levels even in the face of regulation phaseouts, regional discrepancy in intensity of exposure relative to the industrial production and source of contamination associated with PFAS exposures and heavy reliance of exposure within occupationally exposed populations and populations, and that exposure in communities adjacent to point-source sources of contamination with a large impact.

Dietary patterns and food consumption habits are also among the primary contributors to the PFAS exposure profile of most individuals [22,23,24,25,26]. Serum PFAS concentrations varied widely by sex, with higher levels in men than in women [18,27,28]. Among studies focusing on PFAS elimination in females, biological factors, such as menstruation and breastfeeding, were associated with lower serum concentrations [28,29,30,31,32,33]. The worldwide increase in PFAS detection in dairy products and infant formula has raised questions about child exposure risks [7,34,35]. Monitoring PFAS levels in pregnant women is now essential for identifying effective methods to reduce potential exposures and for implementing prevention or intervention programs. Human autopsy studies consistently show the highest PFAS concentrations in the liver, kidneys, lungs, brain, and bone with clear chain-length-dependent patterns [36,37]. Due to their environmental persistence and bioaccumulation, which are critical for human health, and the widespread human exposure to PFAS compounds, their toxic mechanisms must be understood [38,39,40]. The liver is a primary target organ affected by PFAS, while mitochondria are particularly sensitive to PFAS exposure. This review will discuss PFAS-induced alterations in cellular and molecular mechanisms, including mitochondrial function, that lead to liver damage in exposed individuals.

While previous reviews presented PFAS toxicity within the wider context or targeted specific endpoints (e.g., reproductive toxicity, immunotoxicity, or environmental fate), our review gives a complementary and integrated perspective on hepatic toxicity mechanisms. We present a synthesis of recent progress on three major topics: (1) the mechanistic elucidation of PFAS bioaccumulation via protein–phospholipid interactions as opposed to classical lipid partitioning, (2) the data from high-tech 3D cellular models combined with in vivo and epidemiological evidence to link experimental and human health impacts, and (3) the reconciliation of perceived discrepancies in cholesterol metabolism using context-specific and receptor-dependent perspectives. We also highlighted the recent literature (2022–2025) that focuses on environmentally relevant exposure levels and chronic exposure models, addressing the limitations of previous high-dose acute toxicity studies.

## 3. Toxicokinetics and Bioaccumulation

Understanding how PFAS compounds enter the body and distribute through other organs is fundamental to understanding their toxic effects [36,37,41]. PFAS may be absorbed by the intestine when ingested through food. In rats, absorption rates of PFAS compounds can reach 100%, indicating efficient absorption by the digestive system. Indeed, large-scale animal studies demonstrate that the vast majority have absorption rates exceeding 95% and rapid gut absorption of PFAS compounds [36,41,42,43,44,45,46]. Near-complete oral absorption of PFBS, PFHxS, and PFOS has been confirmed in both sexes of Sprague-Dawley rats [44]. Dairy cows fed naturally contaminated feed rapidly secrete PFBSs, PFHxS, PFOS, and PFOA into milk [47], suggesting efficient hepatic uptake based on the ability of organic anion-transporting polypeptides (OATPs, particularly OATP1B1 and OATP1B3) [45,48] and sodium taurocholate cotransporting polypeptide (NTCP) [49], resulting in active secretory transport to milk. Such inactivation is accompanied by enterohepatic recirculation, in which PFAS are secreted in bile through efflux transporters and subsequently reabsorbed by specific uptake transporters into the intestine [50]. Portal-mediated uptake of PFAS by intestinal epithelium exploits channels typically involved in bile acid recirculation, including (1) the intestinal uptake (i.e., OATP2B1) (apical membrane) [48] and functional apical sodium-dependent bile acid transporter (ASBT) mediating active absorption from intestinal lumen [49], (2) the hepatic uptake (i.e., OATP1B1, OATP1B3 [48] and NTCP [49]) mediating an efficient extraction from portal blood into hepatocytes, (3) the renal transporters (i.e., OAT1, OAT3 (basolateral secretion) and OAT4 [51], URAT1 (apical reabsorption)) modulate urinary excretion, and, finally, (4) the amphiphilic nature of PFAS (characterized by perfluorinated carbon chains and acidic head groups) presents high-affinity binding to the organic anion transporters that evolved for endogenous bile acids and organic anions.

After absorption, detailed studies have shown how PFAS compounds cross the intestinal barrier into systemic circulation, bind to circulating proteins, including albumin and low-density lipoprotein, and distribute to extraintestinal organs via the blood [36,37,41,42,52]. Greater than 99% of PFAS components are associated with serum proteins [53]. Such extensive protein binding has profound implications for distribution and elimination kinetics. The structure–activity relationships for the binding selectivity of PFAS compounds to plasma proteins are established. PFAS with carbon chain lengths of seven or more carbon atoms have a higher binding affinity for globulins than short-chain PFAS, which selectively bind to albumin [53]. This selective binding of human serum proteins increases the likelihood of distribution to tissues and the extracellular plasma [54]. In addition, PFAS molecules preferentially bind to cellular phospholipids, which has a marked influence on tissue distribution, especially for longer-chain PFAS [54]. These binding properties play a direct role in determining organ-specific accumulation patterns for PAFS, which accumulate with the highest concentrations in the liver, kidneys, and lungs [36,55,56,57,58,59]. Due to the high protein-binding affinity of these compounds, the protein-rich hepatic tissue makes the liver the primary site of PFAS accumulation [40,59,60]. Per- and polyfluoroalkyl substances (PFAS) demonstrate widespread bioaccumulation in biota and the environment owing to their persistent chemical structures. Traditional bioaccumulation metrics, such as octanol–water partition coefficients (KOW), are used extensively for hydrophobic organic contaminants but are of limited predictive value for PFAS because of their amphiphilic structure and strong affinity for proteins and phospholipids rather than neutral lipids. Meta-analysis of published bioconcentration factors (BCFs) and bioaccumulation factors (BAFs) shows that for aquatic species, legacy PFAS such as perfluorooctane sulfonic acid (PFOS) and perfluorooctanoic acid (PFOA) exhibit substantial accumulation in whole-body tissues, with median log BAF values of 3.55 and 2.16, respectively, in fish, underscoring their capacity to partition into organisms relative to water exposure levels [61]. In Pimephales promelas (fathead minnows), controlled uptake experiments show that accumulation is chain-length-dependent and that both tissue and plasma concentrations of longer-chain perfluoroalkyl carboxylic acids (PFCAs) and sulfonates (PFSAs) increase, with steady-state BAFs up to 26.6 L kg^−1^ for PFOA, and blood–water partitioning coefficients (PBW) as high as 85.8, indicating preferential PFAS partitioning into biotic phases relative to water [62]. In terrestrial plants, PFAS uptake is predominantly via root absorption from soil, where longer-chain PFAS are preferentially retained in root tissues and yield higher bioaccumulation factors when expressed across plant organs; shorter-chain PFAS distribute more readily to aboveground tissues [8]. In vitro partitioning studies demonstrate that PFAS partition into biological compartments is driven by specific affinities for proteins (e.g., human serum albumin) and phospholipid membranes rather than classical hydrophobicity (log KOW), with protein–water distribution coefficients often exceeding membrane partitioning at lower exposure concentrations [63,64]. Long-chain PFAS, including PFOS and PFOA, have higher bioaccumulation potential than short-chain analogs [61,62], and despite low KOW values, can surpass regulatory limits. These protein–water and membrane–water partition coefficients provide biologically relevant analogs to KOW and help explain how PFAS bioaccumulate despite weak neutral lipid partitioning, making experimentally derived BCFs, BAFs, and biological phase partition coefficients more accurate descriptors of PFAS bioaccumulation behavior [64].

In vivo studies reveal that PFAS distribution is primarily influenced by the structure of their carbon chains. Short-chain PFAS compounds accumulate in certain organs on a localized scale, while long-chain PFAS compounds are found more evenly throughout the body [65]. Also, species, sex, and the administered dose level strongly influence PFAS chemical distribution [65]. This distribution is important to understand for determining the different levels of toxicity of PFAS compounds.

PFAS compounds break down slowly because of their strong carbon–fluorine bonds [66,67]. Their half-life in biological systems is directly linked to their carbon chain length. Short-chain variants last for hours; longer chains last for years or even decades [41,65,68]. Machine learning models predict median human half-lives of several years for C8–C11 PFCAs/PFSAs and much shorter durations for short-chain compounds [69]. At the ecosystem level, environmental and food-web studies further demonstrate that PFAS bioaccumulation occurs across trophic levels, with measured bioaccumulation factors in plants, invertebrates, and fish supporting chain-length-dependent accumulation and trophic magnification for several long-chain PFAS [8,70]. These findings support mammalian toxicokinetic data by providing quantitative accumulation metrics across biological systems.

Similarly, biological sex and species differences also play important roles in the elimination half-lives of PFAS compounds [36,71,72]. In a previous study, Dzierlenga et al. found that female rats have shorter elimination half-lives for PFOA and perfluorohexanoic acid (PFHxA) than male counterparts, suggesting that PFAS clearance from the body may be sex-dependent [71]. Chang et al. also reported that perfluorobutanoic acid (PFBA) removal was faster in rats than in mice, suggesting interspecies variability regarding the toxicokinetics of these compounds [36,72]. Nevertheless, both studies used only rodent models of PFAS metabolism, which may not be representative of human PFAS metabolism, given differences in renal transporter levels across species. This limitation significantly widens the existing knowledge deficit and limits the ability to evaluate human health hazards.

There is a big knowledge gap related to novel PFAS groups, raising doubts about the unique metabolic behavior and persistence profiles of new PFAS subtypes, which are not well characterized [73], because most studies have centered on classical perfluorocarboxylic acids or PFCAs and perfluorosulfonic acids [66]. These new PFAS compounds raise questions about elimination kinetics regarding their biotransformation and metabolite production. The biochemical transformations of PFAS precursor compounds and intermediate metabolites are particularly important in biological systems [36]. Fluorotelomer alcohols (FTOHs) and polyfluoroalkyl phosphate esters are volatile precursors of PFOA compounds that can undergo metabolism in humans to yield PFOA and other PFCA compounds [36]. Similarly, several precursors and intermediates of PFOSs (perfluorooctane sulfonamidoethanols or perfluorooctane sulfonamidoacetic acid) undergo biochemical conversion, which ultimately contributes to total PFOS levels in biological systems [36,74,75].

The transformation of PFAS precursors and intermediates is facilitated by various cytochrome P450 (CYP) isoforms, including variants CYP2A6, CYP2C9, the CYP2C19 isoform, and others in human liver microsomes [74,75], though the molecular mechanisms responsible for these transformations are still insufficiently characterized. A striking observation is the lack of successful biotransformation mechanisms for removing terminal PFAS compounds in humans or other mammalian populations [76,77]. PFAS products are excreted from biological systems through limited routes, leading to their persistence. PFAS compounds are excreted mostly in urine and through bile, breast milk, and menstruation [76]. However, their involvement in enterohepatic circulation pathways has led to particularly limited biliary elimination of PFAS’ age [76]. The enterohepatic circulation process, driven by bile acid reabsorption, returns PFAS compounds to the intestinal system and re-enters liver tissues, thereby contributing to persistent increases in PFAS concentration within the liver [50]. Additionally, PFAS bind to serum albumin and globulin proteins results in urinary excretion rates of less than 0.1%, indicating that filtered PFAS compounds undergo considerable reabsorption in renal tubular structures [53,76]. This highly efficient reabsorption mechanism preserves PFAS compounds in the body, possibly responsible for their markedly extended elimination half-lives and potential for bioaccumulation [77,78]. More generally, rodent models highlight the underlying mechanisms, though human-relevant systems require more detailed applications. Potentially, advanced in vitro liver models or human pharmacokinetic studies are needed to address important gaps in PFAS elimination data. The high absorption efficiency, strong protein binding, metabolism resistance, and extensive renal reabsorption result in a toxicokinetic profile characterized by long-term accumulation and continued exposure in target organs, including the liver [36,41,50,53,77,79].

## 4. Major Toxic Manifestations of PFAS

**Figure 1:**
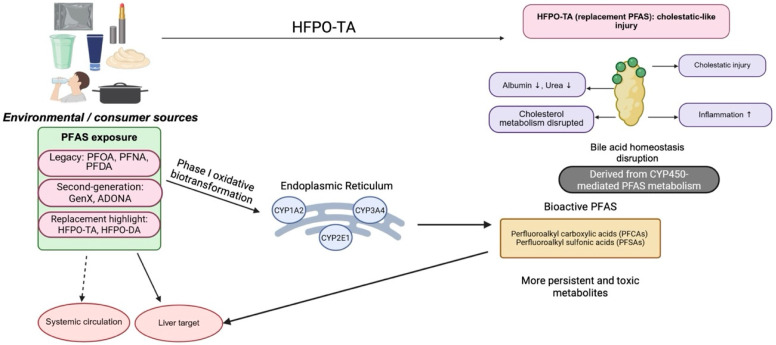
PFAS exposure and hepatic metabolic disruption: schematic representation of the pathways through which environmental and consumer PFAS exposure can impact liver health. The diagram illustrates how PFAS may induce cellular stress (such as ER stress) and undergo metabolic biotransformation, leading to the disruption of bile acid homeostasis and the formation of persistent metabolites.

Prolonged exposure to PFAS in contaminated in vitro systems, animal models, and human populations has established its association with unfavorable hepatic outcomes. Together, PFAS hepatotoxicity presents in three interrelated areas: structural hepatic injury, lipid and bile acid imbalance, and a wider metabolic alteration with glucose and energy homeostasis (Figure 1). These toxic side effects are hepatomegaly, steatosis, hepatic fibrosis, cholestasis, oxidative stress, and hepatocellular apoptosis. Cellular, in vivo, and epidemiological studies suggest PFAS-induced liver damage depends on the structure of PFAS, duration, and dose of exposure. Indeed, hepatotoxicity is associated with toxic responses to PFAS in various organ systems, most significantly the kidneys, liver, and endocrine systems [80,81,82,83], and the liver remains the most prevalent organ system, contributing significantly to xenobiotic metabolism and hepatic bioaccumulation of PFAS molecules [80,81,82,83]. Hepatotoxic effects of PFAS compounds can occur via hepatomegaly and inflammation, oxidative stress induction, apoptosis, bile acid metabolism disruption, and diverse lipid metabolism variations. In vivo cholestatic liver injury, due to bile acid synthesis and transport being disrupted, was demonstrated to be a clinically relevant phenotypic outcome of PFAS-induced hepatotoxicity [36,75,84,85].

Previous epidemiological studies have reported associations with human serum PFAS concentrations and obesity prevalence [81,82,83], suggesting linkage of metabolic dysregulations observed in cell models with population health status. While similar population-level findings do not establish direct causality, the consistency of these data across cohorts supports a meaningful association between PFAS exposure and metabolic dysfunction consistent with experimental observations [81,82,83]. Disruption of hepatic lipid handling is a primary and consistently observed outcome of PFAS exposure among these outcomes. Studies of fatty liver development in response to PFAS exposure have been time- and dose-dependent [86,87,88]. For instance, PFOS has been demonstrated to modulate in vitro specific target genes that encode fatty acid transporters and, in turn, trigger time- and dose-dependent changes in liver fat deposition [86,87,88,89]. Because hepatic lipids are intimately connected to glucose regulation, PFAS-induced disturbance in liver lipid homeostasis often touches on glucose as well. The molecular basis of lipid metabolism disturbance is linked to the increasing incidence of liver disease observed in human populations. The metabolic disruption in PFAS is a matter of closely linked processes that are responsible for glucose metabolism, linking cellular processes directly with a myriad of health consequences. For example, it has been shown that exposure to PFOA significantly elevates fasting blood glucose [90], and it decreases hepatic glycogen and glucose content. These disturbances are mainly determined in the context of metabolic adjustments, which are mediated by enhanced gluconeogenesis and glycogenolysis, and PFAS components have also been found to have functional activities in lipid and liver metabolism in general [91]. The intricate metabolic effects that PFAS exert on glucose metabolism are evident only when new PFAS have been analyzed. In particular, glucose concentrations were elevated in liver tissue after treatment with perfluoro-3,5,7,9-tetraoxadecanoic acid (PFO4DA) or perfluoro-3,5,7,9,11-pentaoxadodecanoic acid (PFO5DoDA) for 140 days. Notably, the serum glucose of the PFO5DoDA-treated group only showed significant increments [92]. This implies that structurally similar PFAS compounds may possess different effects on glucose homeostasis, emphasizing the complex structure–toxicity relationship of PFAS. Exposures, structures, and concentrations of PFAS impact the effects with respect to cell viability, lipid metabolism, bile acid metabolism, glucose metabolism, and amino acid metabolism of hepatocyte cells in many conditions and cell types. These interactions in cellular metabolism characterize the wide range of hepatic pathologies present with PFAS in human beings. These toxic manifestations collectively provide the phenotypic context of our understanding for the interpretation of mechanistic and model-based studies that are examining the molecular and cellular mechanisms where PFAS disrupt hepatic function.

## 5. Major Molecular Mechanisms

**Figure 2:**
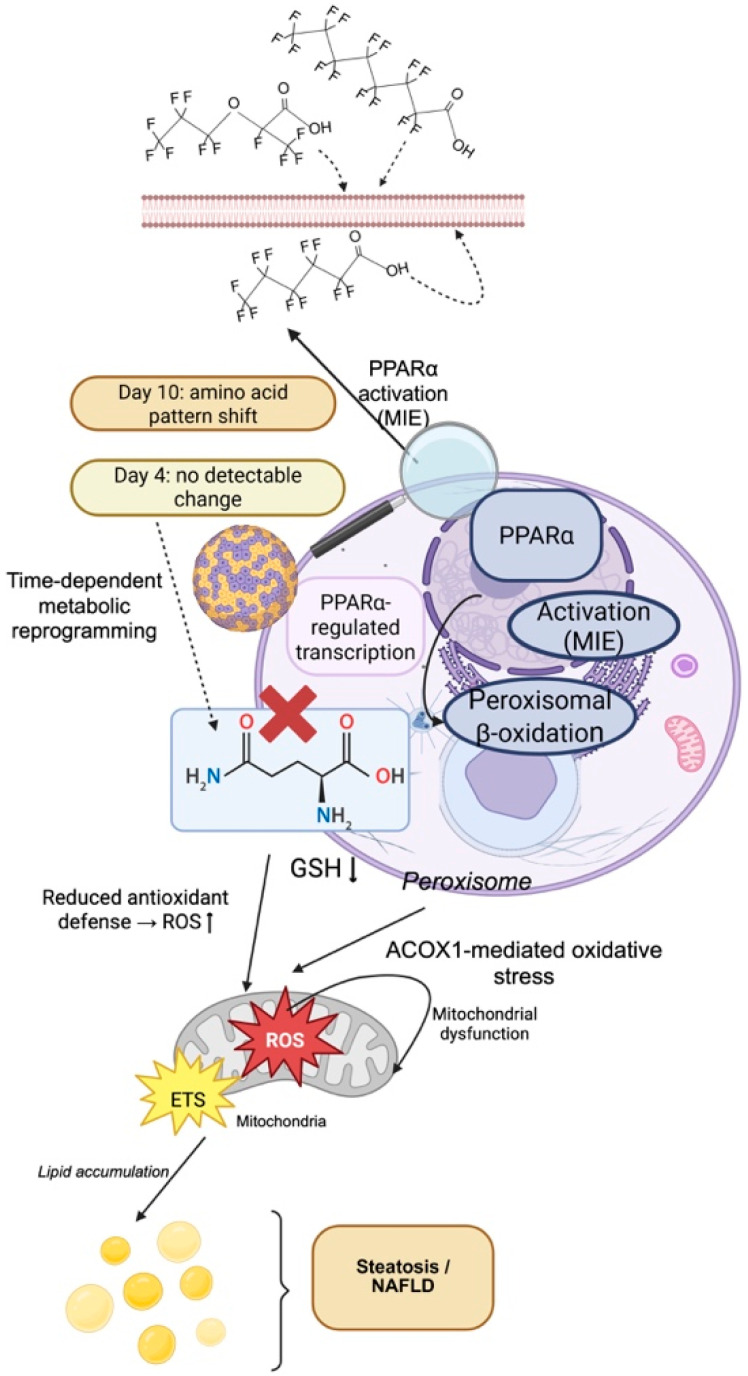
Mechanism of PFAS-induced metabolic toxicity: proposed adverse outcome pathway (AOP) for PFAS-induced metabolic dysfunction. The figure highlights the progression from an initial molecular event (MIE), such as nuclear receptor activation, to downstream effects including peroxisomal oxidative stress, mitochondrial impairment, and altered lipid metabolism. These collective changes result in a state of oxidative stress and time-dependent metabolic reprogramming.

The health effects of PFAS are connected to complex pathways that are important metabolic processes. Understanding these interactions is critical to assessing how PFAS can interfere with normal body functions and be deleterious to health. Nuclear receptors have been identified as the main targets of PFAS compounds, according to researchers. Studies using rodent models indicate that PFOS and PFOA modulate distinct nuclear receptors, thereby promoting the expression of genes involved in metabolism, detoxification, and homeostasis. As one of these targets, the peroxisome proliferator-activated receptor alpha (PPARα) is a key regulator of fatty acid oxidation and lipid homeostasis, which means that its perturbation is particularly important for liver function (Figure 2).

PFOS, PFOA, and PFBS can indirectly inhibit gene expression in sterol regulatory element-binding protein (SREBP) pathways [78,93,94], underscoring the broad impact of PFAS compounds on metabolic regulation. Multiple human nuclear receptors (PPARα, PPARγ, CAR, PXR, FXR, LXR) are directly activated by both legacy and replacement PFAS in reporter-gene and primary hepatocyte assays [95,96,97]. Transcriptomic dissection of PFOA-exposed mouse liver confirms that the gene signature is driven predominantly by PPARα and CAR activation [96]. Hepatocyte nuclear factor 4α (HNF4α), a major regulator of lipid metabolism, is therefore of particular interest for research [95,98]. The effect of PFOA in human hepatoma cells was shown to inhibit HNF4α expression in human hepatoma cultures through pathways unrelated to PPARα [99]. These findings suggest that PFAS compounds may affect metabolic homeostasis through multiple pathways and may exert additive or synergistic effects.

PFAS interactions with nuclear receptors are not limited to simple agonist or antagonist effects. Louisse and colleagues demonstrated that PFOA, PFOS, and PFNA modified PPARα pathway expression and triglyceride levels in HepaRG cells [100]. Moreover, research by Behr and co-workers demonstrated that, although PFOA and PFOS did not alter cholesterol levels under their experimental conditions, they did remarkably enhance the biosynthesis, metabolism, and transport of bile acids in HepaRG cells [95]. Further investigation of PFAS compounds also indicated notable inhibition of CYP7A1, a regulatory enzyme for the synthesis of bile acids from cholesterol, and increased bile canaliculi dilation in well-differentiated HepaRG cells due to PFAS compound exposure [95]. Impaired regulation of bile acid homeostasis affects lipid metabolism and the rest of the liver, linking nuclear receptor modulation to organ-level effects. The interruption of diverse nuclear receptor networks dysregulating metabolic pathways causes significant alterations in lipid, energy and bile metabolism in the liver after PFAS exposures, producing systemic involvement of pathogenic effects that contribute to hepatotoxicity consistent with observations in both experimental studies and human studies [36,96,101,102,103,104].

PFAS compounds cause liver damage through multiple interconnected molecular pathways, particularly by disrupting nuclear receptors. These overlapping mechanisms work together to trigger significant liver diseases. Understanding how these molecular-level disruptions translate into actual tissue damage helps explain the connection between PFAS exposure and liver injury. Research efforts have demonstrated links between the levels of PFAS and the onset of various liver conditions, such as nonalcoholic fatty liver disease (NAFLD), liver fibrosis, and cancer. Briefly, associations between perfluorononanoic acid (PFNA) compounds and PFOA and NAFLD development, particularly among women and older adults, have been suggested [105,106]. Consistent with these epidemiological findings, extensive research has also examined the associations between PFAS exposure, liver fibrosis development, and cancer initiation [59,107,108]. The epidemiology highlights structure–activity relationships associated with hepatotoxicity, with different PFAS compounds showing varying patterns of liver effects. PFAS exposure, particularly PFOS exposure, was found to have relatively stronger statistical associations with hepatic fibrosis progression than steatosis development [109]. This suggests that PFAS compounds may have differential targets for liver disturbances, perhaps through unique molecular mechanisms.

Recent studies show detrimental impacts of PFAS toxicological effects on the liver in laboratory cellular models, as well as various experimental animal studies performed in diverse research environments [110,111,112,113]. PFAS’s toxic effects on liver cells are associated with concentration, carbon chain structure, molecular properties, and compound interactions [111,112]. Wen and colleagues showed that PFOA and heptafluoropropoxy propanoate (HFPO-DA) exert dose-dependent toxic effects in HepG2 cell cultures, predominantly at lower concentrations and after prolonged exposure [112].

Studies investigating chain length effects reinforce the connection between structure and activity in PFAS-related liver toxicity. Ojo et al. found a significant correlation between PFAS chain length and hepatotoxic potential, with longer PFAS chains demonstrating greater toxicity. This relationship, however, was predominantly based on controlled laboratory models and may not be accurate for whole organisms that may use many different metabolic and compensatory pathways [111]. Hence, the extent to which these data may be applicable to human health risk assessments should be carefully evaluated, particularly based on variations in PFAS-binding proteins and metabolism across species. The complexities of PFAS-related liver toxicity extend beyond individual compound effects; the potential interactions between PFAS constituents also add complexity. When combined with other PFAS, particularly PFOA [111,114], it causes additional toxic effects. These cumulative effects are often greater than a single exposure to individual PFAS compounds, which shows that exposure to several PFAS compounds in the environment might carry more risk than single-compound studies would indicate.

These hepatotoxic effects through molecular pathways are intimately related to the nuclear receptor disturbances described earlier. When the peroxisome proliferator-activated receptor alpha (PPARα) pathway is interrupted, hepatic lipid metabolism is fundamentally altered [81,95,100,115]. The direct association between molecular targets and metabolic dysfunction has also been demonstrated by Louisse and colleagues, who reported that PFOA, PFOS, and PFNA remarkably changed the gene expression of the PPARα pathway and increased triglyceride levels in HepaRG cells [51]. The disturbance of bile acid homeostasis is also another important pathway connecting molecular effects to tissue pathological states. For instance, one study demonstrated that while PFOA and PFOS did not increase cholesterol content, they did markedly enhance the production, metabolism, and transport of bile acids in HepaRG cells [95]. Table 1 summarizes the primary molecular targets affected by PFAS exposure, their physiological roles, the toxic outcomes resulting from their disruption, and supporting references from the reviewed literature.

## 6. Advanced Cellular Models and In Vivo Hepatotoxic Effects

In recent years, scientists have been utilizing more complex experimental approaches in order to elucidate the hepatotoxic mechanisms identified in preclinical and clinical settings. Such advanced models are particularly valuable for integrating chronic exposure, multi-pathway disruption, and tissue-level responses that cannot be fully captured by isolated molecular or short-term experimental approaches [84,116]. These complex models provide critical insight into how disruptions in nuclear receptors and modifications in metabolic pathways give rise to tissue-level failures. Especially, 3D hepatocyte culture models have gained traction to investigate the multifaceted effects of PFAS compounds on liver metabolism. These systems address limitations of conventional monolayer cultures by preserving metabolic zonation, prolonged exposure responses, and intercellular signaling relevant to chronic PFAS toxicity. They also more closely resemble Zhang, and others established different metabolite profiles for advanced 3D L-02 cell spheroids exposed to PFOA for 4 or 10 days. Results showed a significant pattern change in six major amino acids for PFOA exposure in the L-02 spheroids after 10 days, but no detectable change after only 4 days of exposure [116]. The time-dependent response pattern highlights the role of exposure duration in PFAS toxicity. Studies focused on short-term exposure may not reveal the complete metabolic effects. Notably, they also showed that the inhibition of glutamine metabolism pathways diminished glutathione (GSH) production and increased oxidative stress in the L-02 spheroids [116]. The greater complexity of 3D culture models has highlighted vital distinctions in the hepatotoxic potential of various PFAS compounds. Hepatotoxicity comparison of PFOA and hexafluoropropylene oxide trimer acid (HFPO-TA) in advanced 3D primary cell spheroids showed that HFPO-TA exhibited significantly increased hepatotoxic potential [84]. The same study also demonstrated that long-term exposure of primary mouse liver spheroids to the PFOA replacement HFPO-TA profoundly disrupts bile acid homeostasis and induces cholestatic injury [84]. This greater toxicity was characterized by decreased cell viability, increased inflammation, reduced albumin and urea production, and significant disruption of cholesterol metabolism pathways [84]. This metabolic and cellular observation is supported and developed in whole-organism studies that demonstrate the biological importance of the observed molecular and cellular effects. Findings from advanced model systems have also reinforced mechanistic observations reported in earlier in vivo and epidemiological studies. The role of the PPARα protein as a primary molecular target of PFAS-induced lipid metabolism disruption has been well described throughout the literature [81,115,117], suggesting a mechanistic connection between lipid microenvironmental observations and molecular/whole-organism metabolic influences. Attema and co-workers reported that HFPO-DA and PFOA enhanced lipids in the liver through the PPARα pathway, and in PPARα-deficient mice, lipid levels were much lower [96]. This finding demonstrated the central role os PPARα in PFAS-related hepatic lipid accumulation. Nevertheless, despite their increased physiological relevance, advanced cellular and animal models remain limited by species-specific metabolic differences and the absence of fully integrated endocrine and immune signaling, underscoring the need for cautious extrapolation to human health outcomes [80,81,115,117]. Table 2 provides a comprehensive overview of experimental model systems used to study PFAS hepatotoxicity, including the compounds tested, key experimental findings, mechanistic insights gained, and relevant citations.

## 7. Species Differences and Clinical Relevance

Mechanistic understanding from laboratory studies to human health needs to consider that species metabolize, distribute, and respond to PFAS very differently. These differences extend beyond academic interest and directly influence risk assessment and regulatory interventions. The most dramatic instance of this variation between species is observed in cholesterol metabolism, which relates directly to the nuclear receptor pathways identified above. PFAS levels correlate positively with total cholesterol concentrations in human populations [87]. In laboratory mouse models, PFAS exposure has been reported to produce variable effects on cholesterol metabolism, including both increases and decreases in serum cholesterol, dependent on dose, exposure duration, and receptor context [96,118]. This apparent divergence between human and animal responses initially complicated toxicological interpretations. However, emerging evidence suggests that these differences reflect context-dependent biological responses rather than a fundamental contradiction between species [118]. Furthermore, clarifying this contradiction shows that different species express signals from different nuclear receptors differently. The PPARα pathway has been found in many studies to be an important mechanism that mediates PFAS toxicity. Indeed, serum levels of PPARα in mice can be about ten times higher than in humans, indicating that the reported cholesterol level differences are species-specific, due to the fact that cells expressing these receptors differ in major components of PFAS action [125]. Recent studies demonstrate that downregulation does not universally characterize cholesterol responses in mice. Under chronic, steady-state PFOA exposure conditions at environmentally relevant doses, serum and hepatic cholesterol levels increased in a PPARα-dependent manner [118]. This is in contradiction to previous studies, which primarily found decreases in cholesterol levels under high-dose or acute exposure conditions [96]. This suggests that the downregulation of cholesterol is not a universal response to PFAS exposure in mice, but rather a reflection of specific physiological and environmental conditions.

Such mechanistic insights were corroborated by work with genetically modified animal models that closely resemble human receptor expression profiles. In previous work by Schlezinger and others, researchers observed substantial changes in serum cholesterol for PFOA in PFOA-exposed PPARα-humanized mice, which provides evidence for the association between PPARα expression patterns and species response to PFOA [86,118]. This suggests that while the basic toxicity mechanisms are similar between species, the direction and severity of effects may differ because of differences in receptor expression and metabolic pathways after PFAS exposures.

What makes these mechanistic insights particularly valuable for clinical practice is that they are backed up by studies measuring biomarkers of liver and bile acid metabolism. A study reported cholestasis assessed by increased activity of serum gamma-glutamyl transpeptidase (GGT), higher cholesterol and bile acid levels, and elevated liver risk factors in mice after exposure to a blend of five PFAS: PFOS, PFHxS, PFOA, PFNA, and HFPO-DA [85]. These in vivo observations are similar to those seen in human studies and merit the clinical significance of mechanistic investigations. Indeed, a study by Guo et al. found that PFAS exposure caused alterations in mRNA and protein expression of enzymes that are essential in liver bile acid metabolism, through extensive RNA sequencing as well as Western blot analysis [110]. The consistency in these molecular alterations between different experimental conditions and their relationship with human analog biomarkers emphasizes the clinical significance and implications of what has been observed.

Species differences extend beyond endpoint comparisons, involving key aspects of PFAS toxicokinetic characteristics. PFAS compound distribution is strongly affected by species, biological sex, and dosage parameters of compound exposure [65]. These differences have a direct bearing on the intensity of response and timing of effects; they also affect species sensitivity to various PFAS compounds. Understanding these species differences is critically important to interpret toxicological data and include them in risk analysis for human health. The mechanisms of PFAS toxicity show similarities among species, but quantitative responses vary significantly. Thus, mechanistic data from animal models could significantly help however human relevant exposures have to be conducted and taking into consideration while making regulatory decisions.

## 8. Function Impairment and Energy Metabolism

It is widely acknowledged that the dysregulated cellular energy metabolism is a key mechanism involved in the detrimental hepatic effects of PFAS compounds. Liver depends on mitochondria for β-oxidation of fatty acids, oxidative phosphorylation for ATP, and maintenance of redox homeostasis. Exposure to PFAS drastically disrupts these processes, leading to a cascade of energy shortages and oxidative injury, causing development into clinical liver diseases such as non-alcoholic fatty liver disease (NAFLD)/metabolic-dysfunction-associated steatotic liver disease (MASLD). Impaired fatty acid β-oxidation (the major mitochondrial route for lipid-derived energy) provides the most unequivocal evidence of PFAS-induced mitochondrial dysfunction. Occupational workers with high exposure to PFAS are also shown to exhibit clear β-oxidation abnormalities, with PFOS emerging as the predominant compound in metabolomics [126]. As such, incompletely oxidized fatty acids, decreased ATP production, and the cell’s energy crisis ensue [126]. In bacterial models (as a proxy for conserved mechanisms), PFOS and PFOA are able to induce membrane disruption, a significant amount of reactive oxygen species (ROS) production, and DNA damage, hence proving that basic mitochondrial toxicity pathways are conserved between species [127]. PFAS induce excessive mitochondrial ROS production, surpassing antioxidant mechanisms, including glutathione and superoxide dismutase. The oxidative stress induces lipid peroxidation, protein carbonylation, and mitochondrial DNA damage in hepatocytes [127,128,129]. This imbalance in redox responses is chain-length dependent: those longer-chain PFAS (C8-C11) are more effectively damaging to mitochondrial membrane potential and electron transport chain efficacy than the shorter-chain compounds; however, some shorter-chain compounds tend to elicit compensatory responses in some models [69]. Bioenergetic collapse is also prominent. PFAS uncouple oxidative phosphorylation, attenuate the proton gradient, inhibit mitochondrial biogenesis, and alter the mitochondrial dynamics (fusion/fission balance). PFAS mixtures induce endoplasmic reticulum stress in human liver spheroids and primary hepatocytes by activating unfolded protein response (UPR), which in turn leads to membrane contraction, mitochondria atrophy, and apoptosis [84]. PFOS specifically affects mitochondrial calcium and iron homeostasis, which drives hepatic insulin resistance, which is an important link between mitochondrial failure and metabolic disease [128,129]. The mitochondrial defects directly play a role in the progression of NAFLD/MASLD, which can be listed as follows: (1) Steatosis is due to inhibition of β-oxidation and modification of lipid handling. (2) Steatohepatitis is the by-product of ROS-induced inflammation and Kupffer cell activation. (3) Chronic oxidative injury and pro-oncogenic signaling encourage fibrosis and carcinogenesis [105,106,130]. Other emerging PFAS seem as potent or even more potent in causing mitochondrial toxicity. One such example is HFPO-TA (a GenX processing aid), which significantly disturbs bile acid homeostasis and induces cholestatic damage in 3D mouse liver spheroids by mitochondrial pathways [84]. These mitochondrial disruptions do not occur in isolation as they translate directly to organism-level metabolic disease. In childhood, mitochondrial-mediated lipid dysregulation has been associated with short-chain PFAS (e.g., PFHpA) [124], associated with MASLD in children. The role of glucose homeostasis only emphasizes that mitochondrial dysfunction is connected with global metabolic dysfunction. PFOS exposure disrupts cholesterol and triglyceride homeostasis in the blood circulation [99], affecting whole-body energy metabolism that contributes to high risk for cardiovascular diseases and type 2 diabetes [108,131]. PFOS attaches to hepatic nuclear receptors, resulting in lipid and fatty acid metabolism and related measures of hepatic injury [96]. These hepatic effects stem from widespread perturbations of glucose homeostasis and perturbations in critical energy substrates like amino acids and purines [108,132]. Mitochondrial dysfunction not only impedes energy production but also negatively affects antioxidant defenders and programmed cell death regulation. Disturbances of iron and calcium concentration, specifically in liver mitochondria, are expected to lead to insulin resistance, and excess oxidative stress and apoptosis limit cellular maturation [128,129,133,134,135]. Placental trophoblast studies have also shown PFOS decreases mitochondrial abundance and activity, both of which support preeclampsia and emphasize the developmental role of the mechanism in more general terms [136,137]. The mitochondrial dysfunction associated with PFAS hepatotoxicity can be viewed as potentially the most convergent multiplier pathway that synergistically combines nuclear receptor dysfunction [96], oxidative stress, lipid deposition, and inflammation to promote PFAS hepatotoxicity. It offers a mechanistic conduit between molecular initiating events and clinical liver disease, and explains what low-level chronic exposure looks like, leading to major long-term risk.

## 9. Emerging PFAS Compounds and Future Health Implications

Mechanistic understanding is only one piece of the puzzle, and meaningful public health protections also depend on regulatory actions that reflect this emerging science. As knowledge of well-known and commonly employed PFAS compounds expands, new challenges emerge from newer PFAS variants, which pose complex challenges for health monitoring and regulatory development. The models developed after studying familiar compounds offer a good basis for the risk assessment of these new materials; however, they also highlight important knowledge gaps. Recent epidemiological studies have confirmed positive associations between PFOA, PFOS, PFNA, and PFHxS and liver injury or disease. Two independent high-exposure occupational cohorts (the C8 Health Project and an Italian fluorochemical worker cohort) both report elevated liver enzymes and increased liver cancer mortality linked to very high historical PFOA exposure [138,139]. Yet, there is little comprehensive work on newer PFAS compounds. This lack of information is concerning, given that molecular pathways elucidated in well-known PFAS suggest that even slight structural changes could lead to varying toxicity patterns while still being activated through similar pathways. Emerging PFAS compounds interacting with environmental and biological systems must respond to the same level of complexity. Interestingly, several molecular studies showed that these novel PFAS compounds exist in food supplies and show considerable liver toxicity [140,141]. Such liver toxicity in new compounds suggests that the liver injury mechanisms observed for established PFAS compounds may also apply to these newer variants, likely through similar nuclear receptor interactions as well as mitochondrial dysfunction pathways.

The regulatory challenges of these new PFAS compounds are significant. The risks associated with exposure cannot be ignored at the regulatory level [142]. Filling the knowledge gaps concerning these compounds will be of utmost importance, and science-based, evidence-based regulation will facilitate actions for improvement [143,144]. The mechanistic understanding developed for classic compounds offers a basis for deciding how to prioritize research toward new variants and appropriate approaches to risk assessment.

Emerging PFAS compounds show diverse structural architectures that can lead to unique toxicological characteristics. Some specific endocrine-disrupting PFAS compounds include C7-C13 perfluorocarboxylic acids, C4-C8 perfluorosulfonic acids, PFAS precursors, and GenX chemicals [140]. Based on the structure–activity relationships of earlier compounds, this may lead to distinct potencies and organ specificities with comparable basic mechanisms. New PFAS compounds pose serious health risks beyond liver damage, including effects on various organ systems in the body. Beyond hepatotoxicity, both legacy and emerging PFAS are increasingly associated with cardiovascular effects, including dyslipidemia, hypertension, and endothelial dysfunction [130]. PFOS, PFOA, and perfluorodecanoic acid (PFDA) have been shown to interfere with the regulation of thyroid hormones [141,142]. Epidemiologically, PFOA exposure is also associated with increased risks of kidney, prostate, and testicular cancers [142], whereas PFOS, PFOA, and possibly PFHxS and PFNA have been linked with significant immune-mediated toxicity [143,144]. PFOA exposure in women has been linked with reduced fertility, preeclampsia, and hypertension during pregnancy [144]. The potential developmental toxicity resulting from PFOA exposure on growth, cognitive function, and behavioral processes in childhood has also been implicated [144].

The health effects of classical PFAS pollutants provide a robust foundation for understanding what new variants can potentially do. These compounds disrupt nuclear receptors and cause mitochondrial problems or changes in metabolic pathways that lead to liver toxicity, which might also affect other organs. Notably, the endocrine-disrupting effects may also involve the same mechanisms as the metabolic changes seen in liver tissues [96,145,146].

Evaluating new PFAS agents is difficult, considering their structural differences and lack of toxicological information. However, what we have learned from studying well-established PFAS will continue to guide how we develop screening methods and direct future research. For instance, the nuclear receptor interactions, mitochondrial dysfunction, and metabolic disruptions that are relevant to PFAS-induced liver toxicity can provide a benchmark for evaluating potential toxic effects of these novel compounds [119,120,147]. Moreover, the structure–activity relationships occurring in common compounds demonstrate that chain length, functional groups, and protein-binding capacity of some of the new variants are likely to be important factors in their toxicity [121,122]. This mechanistic explanation leads to predictive models and enables the choice of acceptable testing systems for the evaluation of novel compounds [148]. When it comes to novel PFAS compounds, coupling this mechanistic information with exposure assessments is particularly important given insufficient epidemiological data [149]. The bioaccumulation and persistence properties associated with long-term exposure of established compounds are likely to be found in many new variants as well. This means that small exposures can add up to substantial body burdens over time.

## 10. Limitations and Future Directions

Although some advancements have been made to our understanding of PFAS hepatotoxicity, our ability to fully predict human health effects in terms of toxic substances and in the evaluation of possible emerging contaminants is limited by several significant limitations. Available in vitro models (e.g., 2D hepatocyte cultures and HepaRG cells) are limited based on the species-specific metabolic divergence and lack of tissue structure to characterize intact liver function [51,123]. Despite more advanced human-relevant receptor systems and the presence of 3D hepatocyte systems more approximate to chronic exposure conditions, these models do not yet reproduce the metabolic interactions, enterohepatic recirculation, and multiorgan crosstalk found in whole organisms. In animal models, interspecies variability in renal transporter expression, receptor activation thresholds, and metabolic enzyme profiles has a substantial role in dose–response interactions and toxicity [57,118,150], and similar limitations apply. However, the most drastic example of this species variability can be found in cholesterol metabolism, where PFAS exposure yields opposite effects in rodents compared to humans based upon PPARα expression patterns, receptor context, and dietary backgrounds [96,118,151]. One key unexplored area is that of PFAS mixtures, which better describe real-world exposure settings than single-compound studies [117,137]. The vast majority of experimental designs have investigated legacy compounds at high doses, but human subjects are exposed to dozens of PFAS at lower amounts in concurrent diets and/or drinking water and consumer products [22,23]. Mixture toxicity evaluations have been limited, especially for mixed mixtures of legacy and recently developed PFAS, which have remained ill defined in their interaction with additive, synergistic and antagonistic effects [117,123]. Moreover, the quick proliferation of chemical replacement has led to thousands of poorly characterized substitutes becoming available for commercial use, many of which are structurally similar to regulated products but lack basic toxicological characterization [152,153,154]. The issue is intensified by sparse analytical methods to analyze and quantify ultrashort-chain and polymeric PFAS, which might have different bioaccumulation and toxicity profiles [154].

Long-term epidemiological studies connecting PFAS exposure to hepatic and metabolic endpoints are required, especially with cohort studies that capture latency periods, sensitive life stages (prenatal and childhood) and cumulative exposures [87,124,127]. Biomonitoring strategy should no longer be limited to legacy compounds and instead include emerging PFAS and transformation products, and biomarker panels with the incorporation of a multi-omics approach will be crucial for subclinical manifestation [110,126]. Research priorities for the future should concentrate on a few aspects. First, the advent and validation of predictive quantitative structure–activity relationship (QSAR) models could lead to rapid hazard screening for emerging PFAS with respect to physicochemical properties and structural features and eliminate the need to perform more expensive toxicology testing on single compounds. Second, identification of such mechanistic biomarkers (metabolic patterns, mitochondrial parameters, and nuclear receptor activation profiles) would potentially help to detect PFAS-induced hepatotoxicity at an early stage and offer translatable endpoints for cross-modality model systems [110,126,131]. Thirdly, population sensitive studies such as pregnant women, infants, those with pre-existing metabolic disorders and occupationally exposed workers are crucial to characterize the risk variables and establish the limits for protective exposure [14,124,127]. Finally, incorporating multi-omics methodologies (transcriptomics, proteomics, metabolomics, and lipidomics) will allow systems-level information on PFAS toxicity and pinpoint convergent pathways that persist across chemical substitutions [110]. By addressing these knowledge gaps through integrated experimental and epidemiological approaches, the scientific community can provide the mechanistic foundation required for the evidence-based regulation and protection of public health.

## 11. Conclusions

The reviewed information describes the main hepatotoxicity mechanisms of PFAS by synthesizing toxicokinetic principles and molecular routes, as well as clinical data, to elucidate the way the long-term environmental pollutants are known to impair liver function. This is where nuclear receptor dysregulation (especially PPARα and CAR) becomes the driving components to toxic metabolic processes that are amplified by mitochondrial dysfunction via oxidative stress, β-oxidation dysfunction, and altered energy metabolism. Species-specific differences in receptor expression, metabolic enzyme profiles, and transporter activity greatly dictate the extent and type of toxicity responses, rendering direct extrapolation from animal models into human health risk assessment difficult. Importantly, the framework developed from legacy compound studies offers us a framework to predict toxic patterns in new PFAS, even though considerable research gaps persist. Although there is an accelerating awareness of the hazardous effects on health and the environment of PFAS, the extant regulatory approaches are fragmented and reactionary. Although the U.S. Environmental Protection Agency has made progress, namely with the PFAS Strategic Roadmap (2021–2024), an expansion of its goals regarding research, restriction and remediation, efforts have lagged behind the pace of scientific recognition of PFAS toxicity [155]. By not acting to change the current maximum contaminant levels (MCLs) for PFOA and PFOS, the agency is using a conservative response that ultimately misses the boat in dealing with the thousands of unregulated PFAS analogs currently used. Proposed rules to clarify supplier notification requirements for PFAS under the Toxics Release Inventory under the Toxic Substances Control Act (TSCA) essentially target manufacturers and not downstream users, thus leaving significant holes in data and accountability. Since there is no single federal standard for compliance, compliance obligations vary by state, which creates regulatory ambiguity for industries and enforcement challenges for policymakers. The mechanistic insights presented here highlight the need for class-based regulation that targets shared structural features as well as toxicological pathways, not thousands of PFAS alone. However, these substitution-based approaches have been ineffective, because new alternatives tend to possess the bioaccumulative nature and hepatotoxicity of legacy chemicals [152,153]. Robust policies must integrate mechanistic insight with epidemiological data to set exposure ceilings, expand environmental monitoring to evaluate new PFAS, and include sensitive populations in risk assessment frameworks. Although the EPA affirmed enforceable standards for PFOA and PFOS in May 2025, the simultaneous extension of compliance deadlines to 2031 and the rescission of regulatory determinations for other PFAS (such as GenX and PFNA) underscore the persistent tension between public health objectives and regulatory implementation. These shifts highlight the urgent need for a management strategy that integrates scientific research, transparent governance, and proactive policies supported by mechanistic toxicology studies to safeguard communities from ongoing PFAS exposure. 

## Figures and Tables

**Table 1:** Key Molecular Targets, Roles, and Associated Toxic Outcomes of PFAS.

Molecular Target	Role/Function	Toxicity Outcome	References
PPARα (Peroxisome Proliferator-Activated Receptor Alpha)	Master regulator of fatty acid β-oxidation, lipid homeostasis, and peroxisomal gene expression	Hepatic steatosis, lipid accumulation, altered triglyceride metabolism, enhanced peroxisomal β-oxidation leading to oxidative stress	[85,86,92,96,116,117,118]
HNF4α (Hepatocyte Nuclear Factor 4 Alpha)	Key regulator of hepatic lipid and glucose metabolism, bile acid homeostasis	Disrupted lipid metabolism, impaired glucose homeostasis, metabolic dysfunction independent of PPARα	[93,95,98,99]
CAR (Constitutive Androstane Receptor)	Xenobiotic metabolism regulator, controls drug-metabolizing enzymes and transporters	Altered metabolic gene expression, compensatory nuclear receptor activation when PPARα signaling reduced	[88,96,97,118]
PXR (Pregnane X Receptor)	Regulates xenobiotic detoxification, bile acid metabolism	Crosstalk with other nuclear receptors, modulation of metabolic outcomes particularly in absence of PPARα	[88,96,118]
SREBP (Sterol Regulatory Element-Binding Protein)	Master regulator of cholesterol and fatty acid synthesis	Indirect inhibition of gene expression, disrupted cholesterol and lipid biosynthesis	[78,93,94]
CYP7A1 (Cytochrome P450 7A1)	Rate-limiting enzyme for bile acid synthesis from cholesterol	Inhibition leading to cholestatic injury, bile acid homeostasis disruption, elevated cholesterol	[95,118]
FXR (Farnesoid X Receptor)	Master regulator of bile acid synthesis and transport	Direct activation leading to altered bile acid metabolism and transport	[88,95,96]
LXR (Liver X Receptor)	Regulates cholesterol metabolism and lipid homeostasis	Activation contributing to cholesterol dysregulation	[88,95,96]
ACOX1 (Acyl-CoA Oxidase 1)	First enzyme in peroxisomal fatty acid β-oxidation pathway	Enhanced activity leading to ROS generation, oxidative stress, mitochondrial compromise	[102,118]
Glutathione (GSH) System	Primary cellular antioxidant defense mechanism	Depletion via glutamine metabolism inhibition, reduced antioxidant defense, increased oxidative stress	[116]
Mitochondrial Complexes I-IV	Electron transport chain components for ATP production	Chain length-dependent inhibition (legacy PFAS: all complexes; second-gen: Complex IV only), leading to neurotoxicity and neurodegeneration	[119,120,121,122]
Bile Acid Transporters	Regulate bile acid synthesis, secretion, and enterohepatic recirculation	Enhanced biosynthesis, metabolism, and transport; bile canaliculi dilation; cholestatic liver injury	[95,100,115,116]
NLRP3 Inflammasome	Innate immune sensor mediating inflammatory response	Activation leading to hepatic inflammation, autophagy-mediated lipid disorder	[112,113]
Albumin and Globulins	Serum protein carriers for PFAS distribution	>99% PFAS binding; chain length-dependent selectivity (C7+ to globulins, short-chain to albumin); determines tissue distribution and elimination	[38,39,40,52,53]

**Table 2:** Advanced Cellular Models and In Vivo Studies.

Model System	PFAS	Key Findings	Mechanistic Insights	References
3D L-02 hepatocyte spheroids (4-day vs 10-day exposure)	PFOA	Time-dependent amino acid metabolism changes; significant alteration in 6 major amino acids only after 10 days; diminished GSH production; increased oxidative stress	Glutamine metabolism pathway inhibition reduces glutathione synthesis, enhancing oxidative stress; exposure duration critical for observing metabolic effects	[118]
3D primary mouse hepatocyte spheroids (long-term exposure)	HFPO-TA vs PFOA	HFPO-TA showed significantly greater hepatotoxicity than PFOA; decreased cell viability; increased inflammation; reduced albumin and urea production; profound bile acid homeostasis disruption; cholestatic injury	Replacement PFAS (HFPO-TA) exhibits enhanced toxicity via cholesterol metabolism pathway disruption and bile acid dysregulation, challenging assumption that replacements are safer	[84]
HepaRG differentiated human hepatocytes	PFOA, PFOS, PFNA	Modified PPARα pathway gene expression; increased triglyceride accumulation; enhanced bile acid biosynthesis, metabolism and transport; CYP7A1 inhibition; bile canaliculi dilation	PPARα pathway drives lipid accumulation; bile acid homeostasis disruption independent of cholesterol changes; nuclear receptor modulation causes organ-level metabolic effects	[85,92,95,100]
HepaRG cells	Various PFAS mixtures	Mixture effects on lipid metabolism exceed individual compound effects; concentration-dependent impacts on cell viability and metabolic pathways	Environmental PFAS mixtures show additive/synergistic effects; real-world exposures may pose greater risk than single-compound studies suggest	[119,123]
HepG2 human hepatoma cells	PFOA, HFPO-DA	Dose-dependent cytotoxicity; effects more pronounced at lower concentrations with prolonged exposure; autophagy-mediated lipid metabolism disorder; NLRP3 inflammasome activation	Low-dose chronic exposure reveals toxicity mechanisms not apparent in acute high-dose studies; autophagy plays key role in PFAS-induced metabolic dysfunction	[112,113]
Wild-type mice vs PPARα-deficient mice	HFPO-DA, PFOA	Enhanced hepatic lipid accumulation in wild-type mice; significantly reduced lipid levels in PPARα-deficient mice; hepatomegaly and inflammation	Confirms PPARα as central mediator of PFAS-induced hepatic lipid accumulation; demonstrates receptor-dependent mechanism	[96]
PPARα-humanized mice (chronic steady-state exposure, 14 weeks)	PFOA	Increased serum and hepatic cholesterol (PPARα-dependent); reduced Cyp7a1 expression; stronger responses in mouse PPARα vs human PPARα; variable triglyceride effects by sex and diet; CAR/PXR activation when PPARα reduced	Chronic PFOA increases cholesterol (contrary to high-dose acute studies); species differences in PPARα expression explain human-rodent divergence; reveals complex nuclear receptor crosstalk shaping metabolic outcomes	[86,118]
Sprague-Dawley rats (time and dose-dependent studies)	PFOS	Altered fatty acid transporter gene expression; time- and dose-dependent liver fat accumulation; near-complete oral absorption of PFBS, PFHxS, PFOS	Demonstrates temporal dynamics of PFAS toxicity; specific gene targets for metabolic disruption; confirms efficient gastrointestinal absorption	[44,89]
Male mice (chronic 140-day exposure)	PFO4DA, PFO5DoDA (perfluoroalkyl ether carboxylic acids)	Elevated hepatic glucose levels (both compounds); increased serum glucose (PFO5DoDA only); suppressed hepatic stress signals; glucose and lipid metabolism disturbances	Structurally similar PFAS show divergent effects on glucose homeostasis; newer ether-based PFAS demonstrate complex structure-toxicity relationships requiring compound-specific evaluation	[92]
Dairy cows (naturally contaminated feed)	PFBS, PFHxS, PFOS, PFOA	Rapid secretion into milk; confirms hepatic extraction and enterohepatic recirculation	Demonstrates real-world bioaccumulation and transfer pathways; confirms hepatic processing and recycling via enterohepatic circulation	[47]
Mouse liver (PFOA-exposed, transcriptomic analysis)	PFOA	Gene signature predominantly driven by PPARα and CAR activation; accelerated gluconeogenesis; increased glycogenolysis; increased fasting blood glucose; reduced hepatic glycogen and glucose	Transcriptomic confirmation of nuclear receptor-driven responses; links PPARα activation to glucose metabolism disruption via gluconeogenic pathway enhancement	[91,96]
A/J mice (environmental PFAS mixture)	Environmental PFAS mixture	Hepato-lipidomic homeostasis alteration; complex metabolic changes reflecting real-world mixture exposures	Environmental mixtures produce distinct lipidomic profiles; reinforces importance of studying realistic exposure scenarios	[117]
Human populations (epidemiological cohorts)	Mixed PFAS (PFNA, PFOA, PFOS, PFHxS)	Positive correlation between PFAS levels and total cholesterol; associations with NAFLD (particularly PFNA and PFOA in women/older adults); stronger association with hepatic fibrosis than steatosis; obesity prevalence associations; increased liver injury susceptibility in children	Population-level data supports mechanistic findings; structure-activity relationships evident (different PFAS show varying hepatic effects); validates experimental observations in human health context	[99,105,106,107,108,109,124]

## Data Availability

No new data were created or analyzed in this study. Data sharing is not applicable to this article.

## Abbreviations

The following abbreviations are used in this manuscript:

3D	Three-dimensional
ACOX1	Acyl-CoA oxidase 1
AFFF	Aqueous film-forming foam
AOP	Adverse outcome pathway
ASBT	Apical sodium-dependent bile acid transporter
ATSDR	Agency for Toxic Substances and Disease Registry
ATP	Adenosine triphosphate
ATP5B	ATP synthase subunit beta
BAF	Bioaccumulation factor
BCF	Bioconcentration factor
CAR	Constitutive androstane receptor
CYP	Cytochrome P450
CYP2A6	Cytochrome P450 2A6
CYP2C9	Cytochrome P450 2C9
CYP2C19	Cytochrome P450 2C19
CYP7A1	Cytochrome P450 7A1 (cholesterol 7-alpha hydroxylase)
DNA	Deoxyribonucleic acid
EFSA	European Food Safety Authority
EPA	United States Environmental Protection Agency
ER	Endoplasmic reticulum
FTOHs	Fluorotelomer alcohols
FXR	Farnesoid X receptor
GGT	Gamma-glutamyl transpeptidase
GSH	Glutathione
HBM4EU	Human Biomonitoring for Europe
HepaRG	Human hepatoma/hepatocyte cell line
HepG2	Human hepatocellular carcinoma cell line
HFPO-DA	Hexafluoropropylene oxide dimer acid (GenX)
HFPO-TA	Hexafluoropropylene oxide trimer acid
HNF4α	Hepatocyte nuclear factor 4-alpha
IP3R2	Inositol 1,4,5-trisphosphate receptor type 2
KoNEHS	Korean National Environmental Health Survey
KOW	Octanol–water partition coefficient
L-02	Human normal hepatocyte cell line (L-02)
LXR	Liver X receptor
MASLD	Metabolic-dysfunction-associated steatotic liver disease
MCL	Maximum contaminant level
MICU1	Mitochondrial calcium uptake 1
MIE	Molecular initiating event
NAFLD	Non-alcoholic fatty liver disease
NHANES	National Health and Nutrition Examination Survey
NLRP3	NOD-like receptor protein 3 inflammasome
NTCP	Sodium taurocholate cotransporting polypeptide
OAT1	Organic anion transporter 1
OAT3	Organic anion transporter 3
OAT4	Organic anion transporter 4
OATP1B1	Organic anion-transporting polypeptide 1B1
OATP1B3	Organic anion-transporting polypeptide 1B3
OATP2B1	Organic anion-transporting polypeptide 2B1
OATPs	Organic anion-transporting polypeptides
PBW	Blood–water partitioning coefficient
PFAS	Per- and polyfluoroalkyl substances
PFBA	Perfluorobutanoic acid
PFBS	Perfluorobutanesulfonic acid
PFCAs	Perfluoroalkyl carboxylic acids
PFDA	Perfluorodecanoic acid
PFHpA	Perfluoroheptanoic acid
PFHxA	Perfluorohexanoic acid
PFHxS	Perfluorohexanesulfonic acid
PFNA	Perfluorononanoic acid
PFOA	Perfluorooctanoic acid
PFO4DA	Perfluoro-3,5,7,9-tetraoxadecanoic acid
PFO5DoDA	Perfluoro-3,5,7,9,11-pentaoxadodecanoic acid
PFOS	Perfluorooctanesulfonic acid
PFSAs	Perfluoroalkyl sulfonic acids
PPARα	Peroxisome proliferator-activated receptor alpha
PPARγ	Peroxisome proliferator-activated receptor gamma
PXR	Pregnane X receptor
QSAR	Quantitative structure–activity relationship
ROS	Reactive oxygen species
SIRT1	Sirtuin 1
SREBP	Sterol regulatory element-binding protein
TFA	Trifluoroacetate
TFR2	Transferrin receptor 2
TSCA	Toxic Substances Control Act
UCP2	Uncoupling protein 2
UPR	Unfolded protein response
URAT1	Urate transporter 1
VDAC1	Voltage-dependent anion channel 1
